# Role of Multiple Infections on Immunological Variation in Wild Populations

**DOI:** 10.1128/mSystems.00099-19

**Published:** 2019-05-07

**Authors:** Ann T. Tate

**Affiliations:** a Department of Biological Sciences, Vanderbilt University, Nashville, Tennessee, USA

**Keywords:** coinfection, disease ecology, ecological immunology, evolutionary immunology, systems biology

## Abstract

A central challenge in the fields of evolutionary immunology and disease ecology is to understand the causes and consequences of natural variation in host susceptibility to infectious diseases. As hosts progress from birth to death in the wild, they are exposed to a wide variety of microorganisms that influence their physical condition, immune system maturation, and susceptibility to concurrent and future infection. Thus, multiple exposures to the same or different microbes can be important environmental drivers of host immunological variation and immune priming. In this perspective, I discuss parasite infracommunity interactions and their imprint on host immunity in space and time. I further consider feedbacks from parasite community dynamics within individual hosts on the transmission of disease at higher levels of biological organization and highlight the promise of systems biology approaches, using flour beetles as an example, for studying the role of multiple infections on immunological variation in wild populations.

## Abstract (precis)

A central challenge in the fields of evolutionary immunology and disease ecology is to understand the causes and consequences of natural variation in host susceptibility to infectious diseases. As hosts progress from birth to death in the wild, they are exposed to a wide variety of microorganisms that influence their physical condition, immune system maturation, and susceptibility to concurrent and future infection.

## PERSPECTIVE

The legacy of the reductionist approach in immunology is a suite of well-defined immune pathways and functional phenotypes that have yielded actionable insight into autoimmunity, cancer, and host-pathogen interactions. However, the need to eliminate biological variation in these types of studies to clarify signal from noise has minimized the recognition that important information lurks in the noise. Relative to inbred laboratory model systems, wild animals (humans included) exhibit considerable immunological variation, and yet we know little about where this variation comes from or how it affects infection outcomes, predisposition to assault by rogue self, or other reproductive or developmental traits associated with host fitness. Genetics is undoubtedly an important contributor to immunological variation, but feedback from the environment also substantively influences immunological variation. From plants to insects to humans, individuals encounter microbes before they are even born and are then deluged as they interact with their environment. These microbes may be phylogenetically similar or diverse and may occupy overlapping niches within the host or partition their occupation in completely different tissues or life stages (1). They produce metabolites that can feed or inhibit other microbes and stimulate polarized immune responses that are not always cross-protective. Thus, the infracommunity (2), or within-host community, of microbes is caught up in a network of facilitative and antagonistic interactions that influences the fitness of each individual member as well as the immunological maturation, survival, and reproduction of the host.

**FIG 1:**
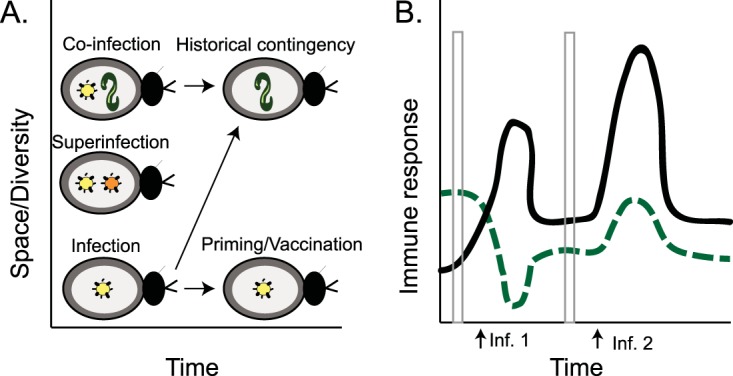
Microbial infracommunity relationships in space and time and their impact on host immunological variation. Hosts can be exposed to multiple parasites that are nearly identical or very diverse (A), and they can superinfect (similar strains) or coinfect (different parasite species) the host at the same time or occupy the host at different times. Even if they do occupy the host at different times, the primary infection can impose an historical contingency effect through its influence on immune system maturation and investment. For example, primary exposure can prime the host immune response to respond more quickly and with greater magnitude upon secondary infection (B) (black solid line). On the other hand, polarization of the immune response during exposure to the first parasite can negatively feed back on an arm of the immune system necessary for defense against the second parasite (green dashed line), resulting in dampened induction of an immune response relative to a host that was not previously exposed. Gray bars highlight the importance of infection history for interpreting immunological variation even in hosts that are not infected at the time of sampling.

To understand the consequences of multiple infections and their impact on host immunological variation in the wild, it is useful to think along axes of both space and time (Fig. 1). For example, developmental constraints on immune system maturation or expression can create variation over time in host susceptibility to a primary parasite (3). Furthermore, a host may become infected and subsequently recover before exposure to another microbe, and yet the immunological imprint of the first infection could impart a historical contingency upon the second. In systems with priority effects (4), the ability of each infracommunity member to colonize, grow, and achieve transmission may depend on whether it arrives before or after other community members. If the timing of exposure is predictable, as with the phenology of plant symbionts (5) or in insects that undergo ecological niche shifts during development (3), then timing could exert powerful pressure on microbial life history evolution as well as the evolutionary dynamics of host immune systems.

Space speaks to the diversity and strength of interactions among microbes that coinfect the host, as competition for energetic resources or physical location can determine the coexistence or exclusion of infracommunity members. For example, mice infected with nematodes were more resistant to *Plasmodium* parasites because the nematodes feed on the red blood cells that the protozoa usually inhabit (1), while cockroaches were more resistant to deadly nematode infection if they were already infected with protozoa that compete with the nematodes for host lipids (6). Multiple infections can influence parameters like susceptibility and disease-induced mortality that are important for dynamics at higher levels of biological organization, including the epidemiology of a pathogen or apparent competition among host community members. For example, African buffalo infected with helminths undergo an immunological shift that renders them more susceptible to tuberculosis-induced mortality (7), such that deworming treatments exacerbate the spread of tuberculosis in the population. If leveraged to capture the interaction of multiple infections in space and time, systems biology approaches provide an opportunity to interrogate holistic patterns of natural variation in disease dynamics from the top down, bridging wild immunology with the reductionist goals of understanding susceptibility to disease.

My research program is taking a systems approach to understand the role of multiple infections in the ecology and evolution of host and microbe life history strategies, including natural variation in immunological investment. We perform experiments on flour beetles (*Tribolium* spp.), agricultural pests that are also the darlings of population biology, hosts to a variety of parasites in the wild, and targets of a suite of “omics” resources in the lab (8). We complement experimental approaches with mathematical models to test our hypotheses and gain inference into the impact of multiple infections on disease dynamics and evolution.

My work on multiple infections started with the observation that flour beetle larvae infected with a common protozoan parasite, the gregarine, sported lower parasite loads after reexposure as adults (9). Since the parasites are cleared from the gut during metamorphosis, this suggests that some aspect of host immunity or physiology remembers the previous infection and primes the host against reexposure. Around the time of this experiment, the phenomenon of invertebrate immune priming was coming into focus, challenging the traditional divide between innate and adaptive immunity. Multiple labs working on bees, beetles, and other invertebrates (reviewed in reference 10) observed that previously exposed individuals, or even the offspring of the exposed, were better protected against subsequent reinfection. Priming is thus an outcome of multiple infections separated by time (Fig. 1) and even generations. We conducted an RNA-seq experiment on our beetles to determine how transgenerational priming with the bacterium Bacillus thuringiensis, a natural beetle pathogen, influenced the offspring response to reinfection. We expected an upregulation in canonical components of the immune system to accompany lower bacterial loads (11, 12). Instead, we found signatures of differentially regulated metabolic and translation processes (10), suggesting that transgenerational priming might be, at least in part, a phenomenon of increased infection tolerance, where priming buffers declining health associated with increasing microbe density.

Immune priming works well in the lab, but is it actually relevant in the wild, when hosts can be coinfected with multiple parasites at the same time? To take a first stab at this question, we collected wild flour beetles that were infected with the ever-ubiquitous gregarine protozoa, taking advantage of natural parasite clearance during metamorphosis. We externally disinfected pupae with bleach to kill sticky oocysts, reexposed half of the emerging adults to the parasites, and then investigated whether the protozoa influenced the strength of transgenerational priming against bacteria. Intriguingly, gregarine-infected mothers were unable to produce primed offspring even as the parasite-free wild mothers bequeathed the primed response (13). Cognizant that the ecological dynamics of wild hosts and parasites could be sensitive to priming, I built a mathematical model that relates the within-host dynamics of resistance and tolerance to epidemiological dynamics in heterogeneous populations. Our model predicts that tolerance to disease-induced mortality can drive up disease prevalence in populations capable of priming (14). This combination of transcriptomic, theoretical, and functional wild immunology brought a dynamical systems perspective to the study of immune priming and raises a suite of new questions about the relative roles of energy, immunity, and epigenetics on the proximate and ultimate dynamics of parasite infracommunity members and their hosts in space and time.

## ON THE HORIZON FOR WILD IMMUNOLOGY AND MULTIPLE INFECTIONS

Many distinct areas of research converge on the study of immunological variation and multiple infections, from the drive to understand the role of gut microbiota on host health to the role of early-life exposure or vaccination on pathogen susceptibility, and even fundamental ecological questions about community stability and trophic cascades. The study of multiple infections is a high-dimensional problem, requiring integration of the number of interacting parasites, their N-wise synergistic interaction possibilities over space and time, the age and stage of the host, and the multitude of ecological feedbacks that play into the framework. Thus far, the systems approach to studying multiple infections in animals has been dominated by cross-sectional transcriptomics, on the one hand, and phenomenological or semimechanistic mathematical modeling of within- or between-host dynamics on the other, providing a disjointed glimpse into the generalizability of the resulting insights. Just as systems biology is reinvigorating the thorny problem of plant priming against repeated herbivory (15), our understanding of multiple infections would benefit from delving more deeply into available systems biology toolkits.

One pressing question for the study of immunological variation is who is infected with what, and when. Charismatic parasites make themselves obvious through disease-induced mortality, but lysogenic viruses and avirulent parasites like gregarines can hide out undetected in lab or wild colonies for generations. We advocate for the use of systems approaches to find these unknown-unknowns and uncover their impact on immunological variation in nature. For example, metagenomic sequencing of focal hosts could help define hidden infracommunity diversity, followed by tailored assays to define natural prevalence by age and stage. A second pressing question is how multiple infections translate into immunological heterogeneity. A single memory T cell arising from a previous infection is likely to have different consequences for subsequent infection dynamics than the polarization of the entire helper T cell subset. Systems biology approaches designed to characterize heterogeneity at different regulatory levels could help reconcile the diversity of phenotypic outcomes associated with multiple infections. In my lab, for example, we are currently employing a combination of proteomics and transcriptomics in our flour beetle system (e.g., reference 12) to resolve the impact of previous and current gregarine infections on the dynamics of inducible immune responses against other natural parasites. Furthermore, we are melding manipulative experiments with metabolomics and mathematical modeling to understand the role of energetic resource competition among the host and its parasites on within-host dynamics and disease transmission. As systems approaches become increasingly available to the wild immunology community, embracing natural variation could stimulate the next sea change in our understanding of the developmental origins of disease and the context-dependent life history evolution of hosts and the organisms that inhabit them.
